# Exploring the Use of a Facebook-Based Support Group for Caregivers of Children and Youth With Complex Care Needs: Qualitative Descriptive Study

**DOI:** 10.2196/33170

**Published:** 2022-06-07

**Authors:** Katherine Jennifer Kelly, Shelley Doucet, Alison Luke, Rima Azar, William Montelpare

**Affiliations:** 1 Health Centred Research Clinic Department of Applied Human Sciences University of Prince Edward Island Charlottetown, PE Canada; 2 Centre for Research in Integrated Care Department of Nursing and Health Sciences University of New Brunswick Saint John Saint John, NB Canada; 3 Psychobiology of Stress & Health Lab Department of Psychology Mount Alison University Sackville, NB Canada

**Keywords:** peer-to-peer support, children, youth, complex care needs, social media, social support

## Abstract

**Background:**

Caregivers of children and youth with complex care needs (CCN) require substantial support to ensure the well-being of their families. Web-based peer-to-peer (P2P) support groups present an opportunity for caregivers to seek and provide timely informational and emotional support. Despite the widespread use of social media for health-related support across diverse patient and caregiver populations, it is unclear how caregivers of children and youth with CCN use and potentially benefit from these groups.

**Objective:**

The aim of this study is to explore the use of a web-based P2P support group for caregivers of children and youth with CCN in New Brunswick, Canada, and investigate factors related to its use by members.

**Methods:**

The study sample consisted of individuals who joined a closed Facebook group and an analysis of content published to the group. In phase 1, a Facebook group was developed in consultation with a patient and family advisory council, and members were recruited to the group. Phase 2 of this study consisted of an observation period during which posts and related interactions (ie, likes, loves, and comments) by members were collected. In phase 3, a web-based survey was distributed, and semistructured interviews were conducted with a subsample of group members. Survey and interview data were analyzed using thematic analysis.

**Results:**

A total of 108 caregivers joined the Facebook group between October 2020 and March 2021. There were 93 posts with 405 comments and 542 associated interactions (448/542, 82.7% likes and 94/542, 17.3% loves). Of these 93 posts, 37 (40%) were made by group members, and 56 (60%) were made by moderators. Of the 108 members, a subsample of 39 (36.1%) completed a web-based survey, and 14 (13%) participated in the interviews. Content analyses of posts by members revealed that inquiry (17/37, 46%), informational (15/37, 41%), and emotional posts (4/37, 11%) were the most common. Emotional posts received the highest number of interactions (median 24.5). In total, 5 themes emerged from the interviews related to the use of the group and mediating factors of interactions between group members: resource for information, altruistic contribution, varying level of engagement, perceived barriers to and facilitators of group activity, and moderators as contributing members.

**Conclusions:**

These findings demonstrate that caregivers of children and youth with CCN seek geography-specific P2P support groups to meet informational and social support needs. This study contributes to the knowledge on how caregivers use Facebook groups to meet their support needs through moderate and passive engagement.

## Introduction

### Background

Children and youth with complex care needs (CCN) are those with multidimensional health and social care needs who may or may not possess a diagnosis of a recognized condition [1]. Children and youth with CCN are present across diverse settings, requiring services from multiple care providers, which can result in significant physical, mental, and emotional pressures on their caregivers. The exact incidence and prevalence rate of children and youth with CCN is not well understood, in part because of ambiguity in the terms used to define this population [2], such as *medical complexities* [3], *special needs* [4], and *medical fragility* [5].

Caregivers of children and youth with CCN face many unique challenges owing to their complicated and multifaceted care needs. Barriers related to financial resources, continuity of care, and service navigation make accessing timely support challenging for these caregivers [6-8]. However, these caregivers possess invaluable experiential knowledge related to the available services and programs, access to resources, and effective professional support. Connecting these caregivers with each other through web-based peer-to-peer (P2P) support is one way to meet their informational and emotional support needs. Social media websites in particular provide an accessible and inexpensive space for the exchange of support between individuals with similar lived experiences.

Web-based P2P support has been shown to provide users with valuable informational, social, and emotional support [9] and allows users to communicate with peers and receive timely support without leaving their homes [10]. Internet-based P2P support allows for connections with peers on the caregivers’ own time [11,12] and improves access despite geographical isolation [13]. P2P support groups present an opportunity for caregivers of children and youth with CCN to learn about and make sense of the maze of services, programs, and treatments available to them as well as the overwhelming amount of information provided to them by various sources [14]. Finally, web-based support allows those facing rare or stigmatized conditions to benefit from web-based discussions with peers [15].

Concerns related to lack of confidentiality and privacy have been identified as barriers to web-based health-related P2P support [16-19]. However, the opportunity to share experiences and connect with peers in similar situations has been reported to outweigh risks related to privacy as well as concerns about web-based negativity and potentially low-quality information [20]. Reaching out to others on the web requires a certain level of candidness and honesty that can sometimes prompt negative support; messages perceived by the receiver as negative or unsupportive are known to lead to poorer overall mental health [21]. Despite the risks associated with sharing personal stories and issues on the web, parents of children and youth with CCN have reported fewer instances of judgment within Facebook support groups than in offline interactions [22].

Many social media websites and applications exist for use by the general public to connect and share content. Facebook in particular has been noted for its use in health-related communication among diverse types of patient and caregiver populations [23-27]. Facebook is among the most popular social networking websites worldwide [28], particularly in Canada [29], where 19.6 million users registered on the website in 2018 [30]. Previous research has demonstrated the prevalence and use of Facebook for health-related P2P support [31], including by parents of children with CCN [22]; however, it is unclear how caregivers of children and youth with CCN use these groups. Moreover, the factors that facilitate the success of these support groups have not been investigated in this population. Understanding the content and interactions between caregivers of children and youth with CCN can inform our understanding of these groups and how they may be leveraged to better support this population.

### Purpose of Research

This research aimed to explore the use of a Facebook-based P2P support group by caregivers of children and youth with CCN in the semirural Canadian province of New Brunswick (NB). Despite previous research demonstrating the use of Facebook groups by caregivers of children and youth with CCN [22], the way in which caregivers use these groups is unclear. Moreover, previous literature has not assessed the factors that contribute to the use of these groups by caregivers. In a preceding environmental scan of Facebook groups for caregivers of children and youth with CCN [32], we determined that there were no province-wide support groups for this population in NB. Therefore, this study aimed to implement and examine the use of a Facebook-based P2P support group for caregivers of children and youth with CCN in NB developed for the purposes of this research.

This research consisted of three phases: (1) developing a Facebook P2P support group for caregivers of children and youth with CCN in NB, (2) assessing its use by caregivers through analysis of posts and interactions (ie, likes, loves, and comments), and (3) exploring the factors that contribute to the group’s activity levels and perceived success or failure by members. The following research questions guided this study: (1) How is the Facebook-based P2P support group used by NB caregivers of children and youth with CCN? (2) What factors affect the activity levels (ie, interactions between members) and perceived success or failure of the Facebook-based support group by caregivers of children and youth with CCN in NB?

## Methods

### Design and Sample

A qualitative descriptive design was used to explore how caregivers of children and youth with CCN used a Facebook-based P2P support group to communicate and to examine factors related to ongoing activity levels within the group. Our sample consisted of caregivers of children and youth with CCN in NB who joined and interacted with the Facebook group and of a subsample of these participants who agreed to take part in the survey and interviews.

#### Phase 1: Development and Implementation of the Facebook Group

A bilingual (English and French) Facebook P2P support group was developed in consultation with NaviCare/SoinsNavi, a patient navigation center for children and youth with CCN in NB. Focus groups and meetings were held with members of the NaviCare/SoinsNavi Patient and Family Advisory Council (PFAC) to delineate an implementation strategy and determine the appropriate content for the group. The PFAC consists of 6 parents or guardians who have children and youth with CCN and 1 young adult who grew up with CCN in NB. Investigators met with the PFAC 3 times during the development of the group and then monthly after its implementation until the conclusion of the study. Specifically, the PFAC informed our team on the development of group characteristics (eg, title, description, membership screening, and rules), plans for discussion moderation and recruitment, and evaluation.

The Facebook group, created for the purpose of this research, was designed to facilitate the exchange of support between caregivers of children and youth with CCN. The group is closed to members, meaning that the member list and information posted within the group are not visible to nonmembers; this was to protect the confidentiality of those within the group and to create a space conducive to the exchange of support. Elements of the group that were visible to nonmembers included the group title, description, and moderators. All prospective members underwent a screening process before gaining approval from the group moderators to join the private group, which included providing informed consent to participate in this research. All members were made aware of the research focus upon joining the group during the study period and were informed when the research observation period ended. Specifically, information about the research was detailed in the group description, screening process of prospective members, and link to a letter of information detailing the research.

The group was moderated by a member of the NaviCare/SoinsNavi PFAC and the NaviCare/SoinsNavi patient navigator. These moderators monitored the discussion page to respond to unanswered posts, ensure the validity of the information, and enforce group rules. Although the patient navigator represented a unique contribution to the support group as a health professional, this individual’s role within the group was simply to offer one perspective in addition to those of the caregivers within the group. The patient navigator’s role was to ensure that the posts received a timely response (ie, respond to posts that did not receive a prompt reply from peers).

The Facebook support group was launched on October 5, 2020. Members were recruited to the group using four strategies: (1) email blast to past and present NaviCare/SoinsNavi clients, (2) media release sent to 35 community organizations that support families of children and youth with CCN in NB, (3) messages sent to moderators of Facebook groups and pages used by caregivers in NB (eg, general parent support groups), and (4) media releases on other social media platforms and websites.

Upon the implementation of the Facebook group, the moderators created a social media plan for ensuring that the group remained active and relevant while it began to grow. The social media plan involved a weekly structure of planned posts that included a welcome post each Friday (tagging all new members that week), a discussion post that prompted members to answer a question or share their experience, and ongoing interaction with posts made by members to ensure that content was not left unacknowledged.

#### Phase 2: Observation of the Facebook Group

Phase 2 of the study consisted of an observation period during which the participants joined and began to use the group; this phase took place over 6 months (October 2020 to March 2021). Content published within the group (ie, posts, comments, likes, and loves) was collected and organized in Microsoft Excel to examine how members and moderators used the group. Additional factors observed to potentially influence ongoing activity levels within the group (eg, time and date of posts) were also noted throughout the research period.

##### Analysis Strategy: Group Posts and Interactions

A qualitative descriptive design was used to investigate the use of the group by members and the factors related to the success or failure of the Facebook-based P2P support group. Specifically, deductive qualitative content analyses were used to analyze the posts published to the group. Content analysis is a qualitative and systematic approach to coding and categorizing text [33] that aims to describe a phenomenon [34]. Posts were categorized according to one of 6 labels based on their content: informational, emotional, inquiry, advertising, fundraising, and other [23,35]. Posts categorized as informational were those containing information of relevance (eg, shared articles or details on a program). These differed from inquiry-based posts, which were centered on a question. Emotional posts described experiences, stories, or narratives. Advertising posts comprised the promotion or sale of a product or service, and posts labeled as *other* were those that did not fit the previous categories. The total numerical count of these posts was recorded along with the total number of associated interactions (eg, likes and comments).

Observed numerical data related to the factors of membership activity (eg, number of interactions) were analyzed using Microsoft Excel. Specifically, descriptive statistics and comparisons related to the frequency of post types (eg, informational, emotional, and inquiry), interactions (eg, likes, loves, and comments), time and date of publication, and source (ie, moderator or group member) were conducted to explore possible associations.

#### Phase 3: Web-Based Survey and Interviews

In phase 3 of the study, a web-based survey was distributed to members within the group, and interviews were conducted. The following section describes the process for data collection and analysis for the survey followed by the process for the interviews.

##### Web-Based Survey: Data Collection and Analysis

The web-based survey was developed using Qualtrics XM (Qualtrics International Inc) and consisted of 16 closed-ended questions and 3 open-ended questions related to the participants’ use of the group and perception of its success or failure. A group administrator posted the survey, available in both English and French, as a link in the Facebook group. The survey questions were developed in consultation with the PFAC and were specific to this research.

Survey results from the closed-ended questions were collated in Qualtrics XM and exported to Microsoft Excel for analysis.

##### Semistructured Interviews: Data Collection and Analysis

The semistructured interviews consisted of 15 open-ended questions based on the participants’ use of the P2P support group, experience as caregivers of a child or youth with CCN, and barriers to and facilitators of using the group to exchange or receive support. Participants were recruited from the closed Facebook group through posts made by moderators. The interviews were conducted in both English and French and were approximately 20 to 25 minutes in length. The interviews took place using Zoom (Zoom Video Communications) videoconferencing software because of its ease of qualitative data collection, data management features, and security options [36]. All the interview participants received an Amazon gift card as remuneration.

The interviews were audio-recorded using Zoom and then transcribed verbatim into Microsoft Word by the lead author (KJK). The interview transcripts and open-ended survey questions were analyzed using thematic analysis [37]. Specifically, the lead author read through the transcripts and assigned initial codes to the content. Codes and associated quotes were collected in Microsoft Excel to produce a summary table [38] and grouped into broader themes using an iterative process to ensure that the original context of the quotes was preserved. Thematic analysis differs from content analysis, which was used to analyze posts from the Facebook group, as thematic analysis aims to provide a comprehensive summary of a phenomenon in the everyday language of those events by remaining close to the *surface* of the words used by the participants themselves rather than attempting to interpret meaning [39]. Previous investigations of web-based P2P support groups that have used content analyses often focus on received support rather than perceived support [40], and the addition of interview data provides an opportunity to better understand and contextualize findings from content analyses [41].

### Ethics Approval

This research was approved by the University of New Brunswick Research Ethics Board (040-2019). A protocol for this research, including the development of the Facebook group, has been published previously [42].

## Results

### Overview

**Figure 1:**
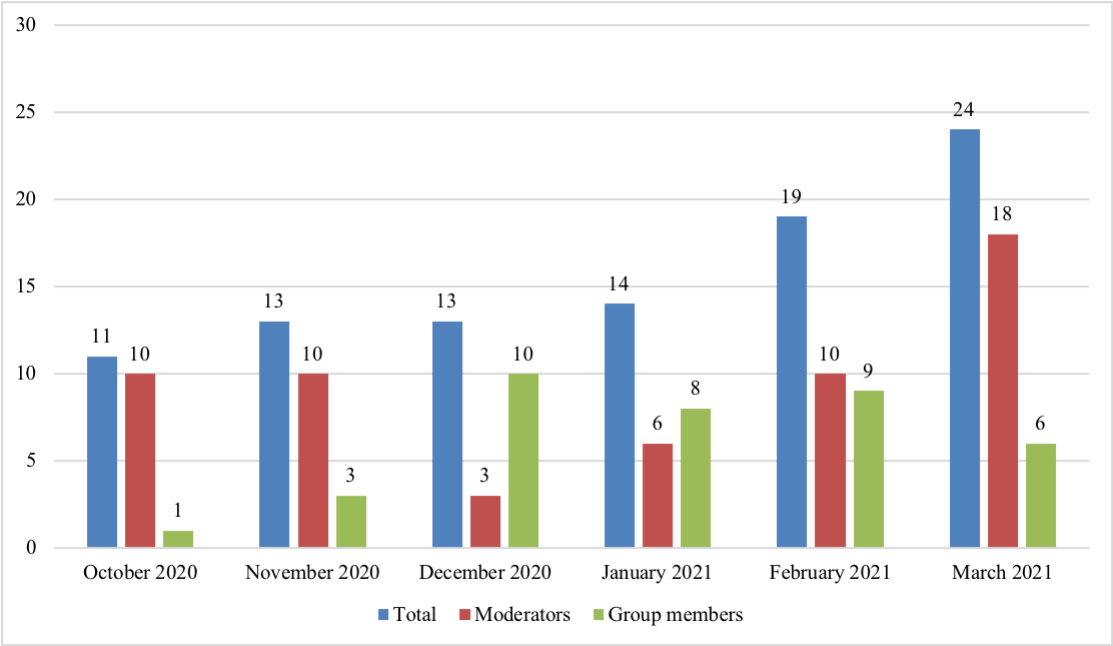
Number of posts published during the data collection period by month and member type.

A total of 108 caregivers of children and youth with CCN joined the Facebook group during the study period. Between October 5, 2020, and March 26, 2021, there were 93 posts with 405 comments, 255 likes (ie, thumbs-up emoji), and 81 loves (ie, heart emoji) from the participants and moderators on the Facebook P2P support group. Of these 93 posts, 37 (40%) were made by group members (ie, caregivers of children and youth with CCN), and 56 (60%) were made by moderators. The date of post publication indicated an increase in the total number of posts each month throughout the data collection period (Figure 1). A breakdown of interactions on posts revealed that most comments, likes, and loves came from group members (537/741, 72.5%) rather than moderators (204/741, 27.5%); specifically, group members made 78.5% (318/405) of the comments, 61.2% (156/255) of the likes, and 78% (63/81) of the loves on posts.

In total, 14 interviews (13/14, 93% in English and 1/14, 7% in French) were completed with members of the Facebook support group. Just over half of the interview participants (8/14, 57%) reported caring for children aged <5 years.

Of the 108 members of the Facebook group, a subsample of 39 (36.1%) completed the web-based survey (all in English). Most of the survey participants were women (29/39, 74%), and the remaining 26% (10/39) preferred not to answer. The survey participants were primarily between the ages of 25 and 34 years (16/39, 41%) and 35 and 44 years (9/39, 23%). Only 10% (4/39) of the participants were aged between 45 and 54 years, and 3% (1/39) were aged >55 years. The remaining participants (9/39, 23%) preferred not to respond. The participants represented a wide geographical range across the province of NB, with nearly one-third (11/39, 28%) reporting the province’s capital (Fredericton) as their place of residence.

More than half of the survey participants (22/39, 56%) reported belonging to the group as members for >3 months. In total, 13% (5/39) of the participants reported a length in membership between 2 and 3 months, and 18% (7/39) reported a length in membership between 1 and 2 months. A total of 13% (5/39) of the participants reported belonging to the group for <1 month.

Most participants reported seeing content from the Facebook group appear on their main timeline a few times per month (12/39, 31%), once a week (8/39, 21%), or once a month (7/39, 18%). Only 5% (2/39) of the participants reported that they had never seen content from the group appear on their main timeline. Most survey participants (29/39, 74%) reported logging in to Facebook daily.

Of the 39 survey respondents, 23 (59%) indicated that they had never published a post within the group; however, when asked about their approximate number of interactions on posts within the group, most participants indicated that they had had 1 to 2 interactions (15/39, 38%) or 3 to 5 interactions (8/39, 21%) with posts. Only 13% (5/39) of the participants indicated that they had never interacted with a post within the group (Table 1). When asked about their perceived comfort with posting in the group, 64% (25/39) of the participants indicated that they felt “comfortable” posting or commenting in the group; those who reported that they did not feel comfortable indicated that their hesitation was due to the research focus of the group (2/39, 5%) and concern that information would become available to personal connections (1/39, 3%).

Nearly two-thirds of the survey respondents (23/39, 59%) reported belonging to at least one other Facebook-based P2P support group related to their role as caregivers of a child or youth with CCN. Most of these participants (14/23, 61%) reported belonging to 3 or more other Facebook support groups. Many survey participants (18/39, 46%) reported using Facebook support groups when they had questions or support needs related to the care of their child.

**Table 1:** Reported number of interactions on group posts by survey participants (N=39).

Interaction frequency	Participants, n (%)
Never	5 (13)
1 to 2 times	20 (51)
3 to 5 times	10 (26)
6 to 9 times	2 (6)
≥10 times	2 (6)

### Content Analysis of Posts

**Figure 2:**
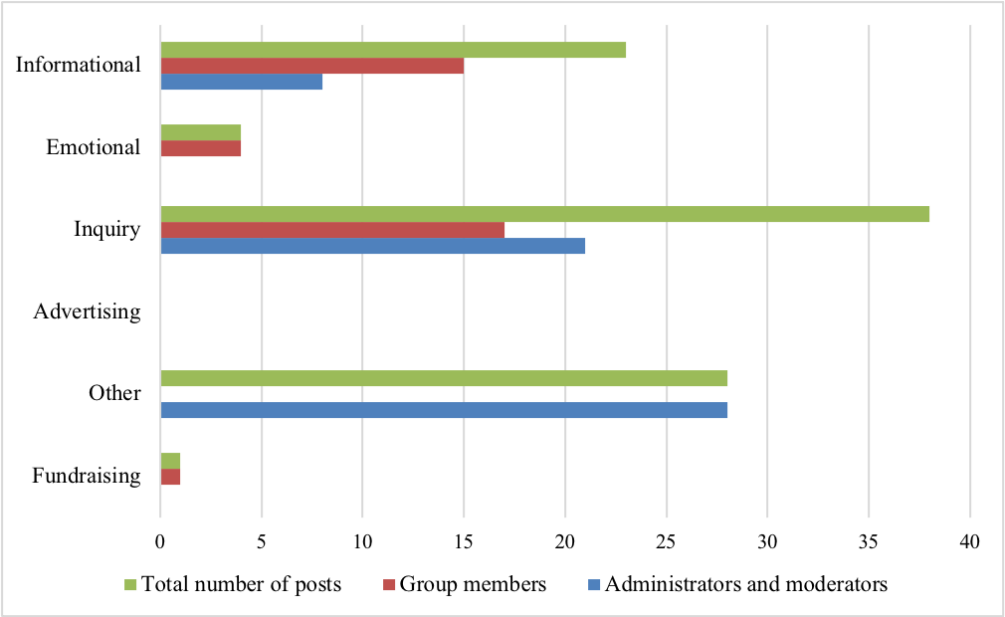
Number of posts according to categorization.

Posts published to the Facebook group’s wall represented 5 of the 6 categories of post types (informational, emotional, inquiry, fundraising, and other); no advertising posts were observed during the data collection period. Combining posts made by both moderators and administrators and group members, inquiry posts were the most commonly observed (38/93, 41%), followed by other posts (28/93, 30%) and informational posts (23/93, 25%). In group members alone, inquiry posts were the most common (17/37, 46%), followed by informational posts (15/37, 41%) and emotional posts (4/37, 11%). Fundraising (1/37, 3%) posts were the least commonly observed type of post (Figure 2).

Posts in the *other* category were published exclusively by moderators and administrators (28/56, 50%). This category consisted of posts welcoming new members (14/28, 50%), invitations for members to introduce themselves or share photos (8/28, 29%), and research-gathering posts (6/28, 21%). The remaining post consisted of an update made to the group description during the data collection period.

Emotional posts received the greatest number of interactions, including comments, likes, and loves (median 24.5, range 18-35), followed by other (median 9.0, range 2-20), inquiry (median 7.2, range 0-29), and informational (median 5.1, range 0-33) posts. Fundraising and advertising received no interactions.

The type of interactions elicited by each type of post varied, with emotional posts (4/37, 11%) receiving the greatest number of comments (median 10), followed by inquiry posts (median 5.5) and other posts (median 4.0). Emotional posts (4/37, 11%) also received the greatest number of likes (n=5) and loves (n=10), followed by other posts (likes: median 4.1; loves: median 1.0) and informational posts (likes: median 2.7; loves: median 0.4).

The time at which posts were made to the group and the number of corresponding interactions indicated a positive but weak correlation between the 2 variables (*r*_97_=0.20, not significant). No correlation was observed between the number of views that a post received and the corresponding interactions (*r*_97_=0.02, not significant).

### Thematic Analysis of Interviews

#### Overview

The use of the Facebook-based P2P support group and factors that affect its perceived success or failure were further explored through a thematic analysis of interviews with group members (ie, caregivers of children and youth with CCN). Five themes emerged from these interviews: (1) resource for information, (2) altruistic contribution, (3) varying level of engagement, (4) perceived barriers to and facilitators of group activity, and (5) moderators as contributing members. Each of these themes is described in further detail in the following sections.

#### Theme 1: Resource for Information

The interview participants reported using the group as a resource for informational support. The participants described other caregivers within the group as a knowledgeable source of information that could assist them in the care of their child or children by providing information gained through lived experience:

And the fact that there is a Facebook group...cause at my age, that’s what they use for information, right? To know that it is from a source that is knowledgeable, and they’ve done their homework and those things, or that they’ll point you in the right direction helps.

Some participants described joining the group in anticipation of support that they would need as their child or children transitioned to new stages, thus using it as a resource for future informational support needs:

I find, for myself, I read comments a lot or I read the post, and then I get a lot of information out of what people are commenting back. I find that is extremely helpful because even if I don’t, if it’s not directly related to me yet, it might be something I encounter later on. So it’s helpful to have, like, “oh, I can go back to this and read it.”

Many participants identified the geography-specific aspect of the group as an important resource for navigational support. Most interview participants indicated that they were members of other Facebook-based support groups that were not specific to NB that aided in their role as caregivers of a child or youth with CCN. These participants described using the NB group to complement support received from their other Facebook support groups; specifically, the NB support group was used for local informational and navigational support needs, whereas many described using condition-specific groups for support related to their child or children's medical care:

The other [condition-specific] groups, I tend to go more for, like, medical things. So for instance, like on my [condition] group, I'll post like, you know, “what medications are you guys being given for seizures and sleep, because we're struggling right now.” And then I can get the support from that. So the other groups, I tend to use more of like a medical piece. But this one here, I see more as like a resource piece, looking for resources and things like that.

Some participants discussed the impact of the COVID-19 pandemic on their role as caregivers of a child or youth with CCN and specifically how it affected their use of Facebook support groups. The participants noted an increase in their use of Facebook for informational support because of additional pressures faced by having a child or youth who was immunocompromised. Moreover, the participants described experiencing barriers related to the pandemic and using the support group to fill the gaps left in their information resources:

I’m so new here and because of the pandemic, it hasn’t really allowed me to go out and explore and find these things for [my daughter]. And all these questions that I’ve asked, everyone’s been very helpful and very kind, and yeah. 

#### Theme 2: Altruistic Contribution

Many participants described using the group to share the knowledge that they had gained through lived experience as caregivers of a child or youth with CCN. The participants described a desire to help other individuals going through similar situations by sharing the knowledge that they had obtained:

So like, now I don’t feel like I’m an expert at all, but I have so many, like, things in my backpack, like that I can reach out to and go to. I wish that I could give that backpack to me eight years ago. Like, other people that I know now that are starting to go through it. And that’s why it’s really awesome that this Facebook group happened, because it’s a way for other people to share with me what’s in their backpack and for me to share with those people.

#### Theme 3: Varying Level of Engagement

The interview participants described a range of engagement with the Facebook group. Although many participants described themselves as *lurkers* and *stalkers* within the group, many still felt that they benefited from participation:

I’m a Facebook stalker, I’m a group stalker, so I just wait for other people to [laughs] post stuff and people have posted exactly what’s on my mind all of the time so I don’t even have to post, which is really nice. Just being on the group.

The participants who reported making contributions to the group through posts and related interactions described using the group infrequently or inconsistently:

I’m not on Facebook a ton. Um, um, so I “like” things and sometimes I’ll share things that I think are relevant to the group. Um, and I have made a post, a post or two and commented on a post.

The participants who reported feeling comfortable posting in the group when they had a question or concern attributed it to the geography-specific nature of the group and observing other members model interactions with content:

I think just personally I still have a hard time putting myself out there. Like, to ask a question. But when I see someone else, like I feel comfortable commenting on someone else, or like, liking and things like that.

#### Theme 4: Perceived Barriers to and Facilitators of Group Activity

Several factors were described by the participants as affecting their use of the group and perception of overall group activity. These perceived barriers to and facilitators of group activity were divided into 4 subthemes: target conditions or diagnoses, research emphasis, privacy of content, and group duration (time since implementation).

##### Targeted Conditions or Diagnoses

Some participants described a lack or low incidence of activity within the Facebook group compared with other Facebook support groups of which they were members. These participants felt that part of the reason for this lack of activity may be the diversity in conditions experienced by caregivers and their children within the group:

Her condition is so rare. I only know of one other family here whose son has [it], like I said so I don’t expect, yeah, I don’t expect to learn too much more about her condition and a lot of the times, her condition isn’t black and white either.

Despite the diversity in conditions, the participants felt that the similarities between the journeys of individual caregivers, owing to the geography-specific nature of the group, may promote the long-term success of the group:

Having [a group based] in New Brunswick has been very helpful, just to know that we can connect to people who are close by. And even just knowing someone is, even if they’re in Moncton, or they’re anywhere else, like just knowing they’re in New Brunswick is helpful, and they’re kind of on a similar journey.

##### Research Emphasis

Some participants identified the research focus of the group as a potential reason for a lack of activity, although these individuals did not feel uncomfortable posting or interacting with posts themselves. The participants who identified the research focus as a potential barrier described it as a unique factor to the group, as an avenue to advocate for gaps in support availability:

I mean I don’t have a problem with it, some people maybe are worried to share things because there’s administrators or moderators in there, you know what I mean? I wouldn’t, but maybe some people wouldn’t complain about services in New Brunswick if they’re worried it would get back to the service provider, I don’t know. I personally don’t think that it makes a difference, knowledge is power, and I think that if people hear what we go through or what our struggles are or what’s lacking or where it’s lacking, that it’s going to help our kids in the long run.

##### Privacy of Content

The private nature of the group and restricting content to members were considered facilitators of activity within the group:

I think that it being, like, a private, like New Brunswick group, um, makes it feel more comfortable.

However, a lack of clarity in exactly what content is visible to nonmembers was identified as a barrier to participation by a participant:

Overall, uh, we haven’t really used it a lot. That’s more because, uh, there’s not the comfort level there, knowing who’s in it and who’s in charge of it, and who can be looking in.

##### Group Duration: Time Since Implementation

There was a sense that the community within the Facebook group was growing. Many participants described referring prospective members to the group as a means to continue building the web-based community. The participants reported feeling that a larger community would lead to increases in group activity, such as more posts and interactions:

Even in like there’s a mom chat group for New Brunswick that’s quite, like, people are constantly posting in it. I think once this group grows like that it will have the same effect, I think, that people will look to that first and they’ll get the support from there.

#### Theme 5: Moderators as Contributing Members

Many participants described the influence of the group administrators and moderators. Most participants felt that the moderators were the primary contributors to the group. When asked about the contributions of the moderators, the participants reported seeing weekly discussion posts intended to maintain activity within the group and interactions with members’ posts. These participants viewed the moderators as active members of the group who interacted with and facilitated discussions:

I think they do a great job because I think see them comment on almost every comment. And, uh, I see that they, they put posts on there, you know trying to facilitate discussion or whatever, which I think is nice too.

## Discussion

### Principal Findings

This study aimed to investigate how caregivers of children and youth with CCN used a Facebook-based P2P support group and explore factors related to its ongoing activity levels. The group attracted a total of 108 caregivers of children and youth with CCN over a period of 6 months upon implementation. Although members only made 40% (37/93) of the posts in the group during the observation period, members in the group were observed to engage with posts a total of 537 times, including 318 comments. These findings are consistent with previous research illustrating that activity within Facebook groups tends to consist of 10 times more interaction with posts (eg, likes and comments) than posts themselves [41].

The survey respondents were mostly women (29/39, 74%); although 26% (10/39) of the participants did not disclose their gender, none reported being men. This is consistent with previous research suggesting that White, female, and college-educated users are more likely to use social media for health-related support than men [43].

Nearly two-thirds of the survey and interview participants (23/39, 59%) reported belonging to multiple P2P support groups related to their role as caregivers of a child or youth with CCN. Many of these participants described using each of those groups for a specific purpose. For example, groups centered on a specific condition or set of symptoms often involved members from all over the world. These groups were considered helpful for informational support related to medical concerns and specific emotional support because of the often rare nature of a condition.

The Facebook group, developed for the purpose of this research, was viewed as an important source of informational support, specifically navigational support for local programs, services, resources, and activities. Most of the survey participants in this study (29/39, 74%) reported using Facebook daily. Previous studies posit that the more intensely an individual uses social media, the more perceived support they receive [44]. The participants in this study reported using the group for these informational support purposes and gaining insight from individuals whom they considered knowledgeable experts. It appeared that the interview participants valued the knowledge available from their peers, which they specifically attributed to the experiential knowledge of their peers [45]. The information obtained from peers within the group included their experience with various services, resources, programs, and activities as well as their opinions and suggestions, which were highly trusted by the participants; this trust in knowledge obtained from peers in similar situations has been previously observed [46].

Content analyses of the posts published to the group showed that inquiry-based posts (ie, those centered on a question) were the most common among group members, followed by informational and emotional posts. Most of the posts published to the group originated from group moderators (56/93, 60%) as a means to promote activity within the group and prevent it from becoming stagnant. However, over the course of the research period, the total number of posts published to the group was observed to increase each month. Despite the short time frame between the implementation and evaluation of the Facebook group, many interview participants also felt that the group was growing in membership and activity levels. Initial recruitment efforts to the group resulted in a corresponding surge in membership, yet membership continued to grow despite the conclusion of the recruitment period. This can be explained by an increase in word-of-mouth referrals made by participants who had joined the group and then shared it with other relevant groups on the Facebook platform.

Emotional posts received the most comments, likes, and loves from group members, specifically receiving the most comments. These posts also received the greatest number of likes and loves, suggesting that group members respond the most to posts based on an emotional support need. As expected, inquiry posts received the next highest number of comments as these posts are generally centered on a question requiring insight from other members and usually develop into a discussion in the comment section.

The use of the Facebook group by caregivers for social support can be explained by the strength of weak ties theory [47]. This theory suggests that social support is exchanged within a social network through strong ties (eg, family and close friends) and weak ties (eg, acquaintances) but that weak ties may be particularly important for eliciting benefits. Where web-based communities with strong ties often result in information saturation, those with weak ties tend to be more heterogeneous and can result in greater access to diverse and stronger information support [48]. Moreover, weak ties can encourage individuals to disclose more personal or sensitive information because of the perception of less judgment [49,50]. Finally, weak ties can be perceived as helpful to individuals seeking informational support to deal with a health issue [51].

The administrators and moderators may have indirectly influenced how caregivers of children and youth with CCN used the group. Previous content analyses of P2P support groups have shown that members seek more emotional support from informal support groups, whereas they tend to seek more informational support from formal support groups led by professionals [52]. One of the explanations for this is that messages posted by trained health care workers are distinctly different from those posted by group members; specifically, messages from trained peer counselors tend to be more structured and detailed than those from peer members [53]. Given the research emphasis and professional experience of one of the moderators, caregivers in this investigation may have viewed the group as a formal support group. However, in a previous investigation of parents of children with special needs, Ammari et al [22] found that parents used geography-specific P2P support groups primarily for informational support needs over emotional support needs because of the collective knowledge of locally available services, resources, and programs among members. Therefore, although it is possible that the moderators influenced the type of support that members sought in this study, previous research supports the notion that geography-specific groups result in the exchange of more informational support.

Many participants expressed a desire to support other caregivers of children and youth with CCN by sharing their own knowledge and experiences. A participant described this lived experience as a collection of knowledge, their “backpack,” which could be shared with those who might be lacking information. Some of these participants expressed feeling compelled to help others, specifically regarding informational and navigational support. Previous investigations suggest that this reciprocity and sharing of knowledge and experiences can help foster friendships and promote positive health behaviors in persons who engage in health-related, web-based P2P support [54]. In this study, sharing one’s experiences was considered an important catalyst for social support.

The interview participants described varying levels of engagement with the Facebook group. Although previous literature suggests that superusers (ie, users that consistently and actively engage with content on social media) are the foundation of activity within P2P support groups [55,56], most participants in this study described themselves as either moderate contributors or lurkers. This was supported by the survey findings, which revealed that most participants had never published a post to the group but had interacted with at least one to two posts within the group. Although many of the interview participants did not actively interact with the content in the group, many described using the group as a source of informational support. Specifically, the participants were often able to find answers to their questions through previous posts or comments, sometimes even using the search bar in the group to see if a topic had been discussed previously. These findings are contrary to previous research suggesting that lurkers do not gain as much from participation in groups as superusers [57] but support the notion that lurkers can benefit from passive interaction with support groups [58].

In this study, the *success* of the Facebook support group was determined by regular use of the group through user-level activity (eg, posts and associated interactions). An overview of the factors related to group activity as identified by the participants is shown in Table 2. Specifically, factors identified by the participants that contributed to their use of the group included the closed privacy designation of the group (ie, content was restricted to members) and the focus on NB caregivers. The geographic specificity of the group appeared to counteract the diversity found between the conditions experienced by caregivers within the group, which was identified as a potential barrier to activity and interactions. Most participants in the survey and interviews reported feeling comfortable posting within the group if they felt the need; those who reported feeling uncomfortable cited concerns related to the research focus. Importantly, only 5% (2/39) of the participants in the study described this as a concern.

The private designation of the Facebook group was an important consideration for attempting to protect the confidentiality of caregivers. Maintaining confidentiality was particularly important in this Facebook group, which consisted of members from a small geographic community. Concerns related to privacy have been identified by patients and caregivers who participate in web-based support [16,17]; however, the benefits associated with sharing such information are considered greater than the potential risks [15]. Privacy concerns related to the use of P2P support forums on social media do not appear to be consistent across all users and may depend on contextual factors [59].

The participants in this study perceived the group to be successful as a place for gathering caregivers of children and youth with CCN and providing a space for the exchange of support. Developing a group that can maintain active interactions among members over a period requires creating a space that is trusted by its members [41,60]. Variables identified in the literature to facilitate trust in Facebook groups include smaller and more homogeneous membership, long group tenure, identity-based groups, and age and gender homogeneity [41]. International diversity, for example, has been negatively associated with trust in Facebook groups [60]. Smaller group sizes with exclusive membership are known facilitators of trust among web-based communities that increase opportunities for new connections within the group [41]. Specifically, groups with >150 members are considered less trustworthy than smaller groups. Apart from the short group tenure, each of these factors was observed in this study of 108 caregivers, suggesting the potential for longevity.

**Table 2:** Identified factors that affected activity within the Facebook group.

	Barriers	Facilitators
Targeted conditions or diagnoses	Group members identified as caregivers of children or youth with a diverse range of conditions or diagnoses, which limited the ability of some members to find disease-specific support.	Participants indicated common struggles and difficulties regardless of condition or diagnosis though all being caregivers of children or youth with CCN^a^.
Research emphasis	The emphasis on research was perceived as a possible deterrent to joining by some members.	The presence of researchers in the group was perceived by some members as a unique factor that could be used for advocacy.
Privacy of content	The “closed” group (ie, group content and membership list was not visible to nonmembers).	Use of content within the group for research prevented some members from posting or interacting with posts.
Group duration: time since implementation	Some members attributed the lack of group activity to the short period since the group’s launch.	Group activity may be associated with group maturity.

^a^CCN: complex care needs.

The findings indicate that moderators were viewed by group members to be active contributors to the group, which then encouraged members to use the group; this supports earlier findings about the importance of moderators for network engagement [61]. Although moderators were perceived as the primary contributors to the group, this interaction by moderators appeared to facilitate group activity. Support groups are moderated by professionals (eg, care providers) [62,63] or peers (eg, other patients or users) [64,65]. This Facebook group was moderated by a patient navigator (care professional) and a member of the NaviCare/SoinsNavi PFAC who has experience being a caregiver of a youth with CCN. A limitation of moderators identified in the literature rests in their ability to answer certain questions from members [66]. Although moderators aimed to respond to posts by group members to ensure posts were never ignored, other group members often provided their unique insight into questions raised. Therefore, although the Facebook group was not solely a P2P support group, as the moderators did not represent the target population, responses from both the moderators and other caregivers integrated to form a unique perspective on issues raised by group members.

The use of Facebook groups to connect patients and caregivers is not without important ethical concerns. Salient among these are concerns regarding the potential to spread misinformation [17] and members’ ability to appraise information [63]. This issue may be less evident in groups that primarily exchange emotional support as these groups appear to exchange fewer posts related to medical information (ie, related to diagnoses, treatments, and medications) [67]. Moreover, research analyzing content in casual information-seeking environments such as web-based P2P groups has shown that the content self-corrects over time as individuals visiting the group validate or correct the posted information [68]. Moderators have been observed to reduce the spread of misinformation [68,69] by enforcing group rules and ensuring that posts remain on topic [70].

### Limitations

The limitations of this research include a time constraint between the implementation and evaluation of the Facebook group and small interview and survey sample sizes. The short recruitment and evaluation periods, for example, may have been a reason for our low overall sample size. This research may have oversampled caregivers who were more engaged in the needs of their children as the survey and interview participants came from a sample of the population who chose to become members of the Facebook group. Moreover, there was overlap between the participants who completed the web-based survey and interviews; specifically, 86% (12/14) of the interview participants also completed the web-based survey. However, the survey and interview questions were different; specifically, the interviews aimed to provide greater context for questions within the survey. The findings from this survey may have also been affected by the modest survey response rate (39/108, 36.1%). Finally, the questions used in the survey and interviews were developed for the purpose of this research and were not validated through a systematic process.

Demographic information was not obtained about members of the Facebook group because of privacy restrictions imposed by the Facebook platform. However, as the focus of the study was on the use of the support group by caregivers of children and youth with CCN, this information was not central to the goals of the study. The survey data suggested that the participants were primarily women (29/39, 74%); therefore, the male perspective is missing from this study. Although we attempted to cast a wide net across the province to recruit participants to the group, it is possible that we may have missed segments of the population, which may affect the generalizability of our findings. For example, the group may have remained unknown or inaccessible to those in rural or remote geographical locations or who are more comfortable speaking in languages other than English and French. Another potential concern is that the research focus of the Facebook group may have affected the way that prospective members approached it and the way it was used by group members. Specifically, concerns regarding privacy may have prevented members from posting content. Moreover, one of the moderators was a patient navigator who did not identify as a caregiver of a child or youth with CCN. It is possible that the presence of a health professional within the group affected the dynamics of the P2P interactions.

Data were not obtained at the user level regarding the number of posts or interactions made by each group member. This was partly owing to the short time span (6 months) of the study period, during which the group experienced a surge in membership. Although prospective members were required to undergo a screening to ensure that the population was restricted to caregivers of children and youth with CCN in NB, this information was self-reported by users and could not be verified by group administrators. As a result, it was not possible to confirm that every member of the group was a caregiver of a child or youth with CCN and lived in the province of NB.

Although the group was developed before the COVID-19 pandemic, the Facebook-based P2P support group was implemented and examined during periods of stay-at-home orders and provincial restrictions. Caregivers of children and youth with CCN were particularly affected by social distancing measures, which led to increased caregiver stress and loneliness [71]. Moreover, disruptions in communication with health care providers and the risk of COVID-19 exposure led to increased stress among caregivers [72]. The unique circumstances presented by the pandemic may affect the generalizability of the findings of this study. For example, it is unclear whether caregivers would have used the group to the same extent outside of the parameters of such extreme circumstances. Many of the interview participants expressed concern about interacting with individuals from outside of their household because of their child or children's immunocompromised conditions; it is possible that these participants may have leaned on support from groups such as the one studied to fill in missing support. It is also possible that the COVID-19 pandemic will change the future use of P2P support groups for health-related communication on social media. The Facebook group, developed for the purpose of this research, continues to operate as a source of support for caregivers of children and youth with CCN in NB. Moderation of the group has been taken over by existing membership, and the group continues to welcome new members.

### Future Work

The Facebook group was identified as an important source of information as well as social interactions by caregivers in this study. Given the close proximity in geographical location between the caregivers in the Facebook group intervention, it is possible that some members of the Facebook group may express a desire to meet face to face at the conclusion of the COVID-19 social distancing measures. Although the caregivers in this study served as a source of information for members of the Facebook group, future research might examine the differences between web-based and offline social support when individuals initially connect on the web. For example, offline social support may result in additional instrumental or tangible support to complement the action-facilitating support exchanged on the web [73]. Moreover, future work might examine how web-based and offline social support networks may influence one another.

Despite the initial uptake of the Facebook group by caregivers of children and youth with CCN, few members reported regularly posting and engaging with content in the group. Previous work on the participation of web-based community members has noted a 1-10-90 pattern wherein 1% of members create 90% of the posts and 10% of members interact with those posts [57]. Although there appeared to be greater participation with content by caregivers in this study, the sample size was small. More research is needed on the presence of lurkers in web-based P2P support groups to better understand their experiences and possible barriers to participation.

One of the eligibility requirements for joining the P2P support group in this study was that members reside in NB, Canada. Through our observation of the group, we concluded that this factor may have created an environment for the exchange of primarily informational support, which is consistent with previous findings [22]. Freedom from geographic constraints is a benefit of using web-based P2P support compared with in-person support groups; however, the degree to which groups are limited by geography appears to affect how a group may be used for support. Future work is needed to determine how the self-reported geographical location of participants affects participation in web-based groups, particularly in comparison with condition-specific groups free of geographic constraints.

Considerations of patient- and caregiver-level characteristics and how they may influence the type of contributions made to groups is also needed. For example, individuals facing a new diagnosis versus those with more experience may use web-based support groups differently [70]. This study found that some caregivers intended to use the group as they faced transitions (eg, school or respite care). Future research might consider examining the experience of caregivers at various points in their care journeys; moreover, these differences in contributions may point to distinct needs between patient and caregiver populations. Future work may also consider determining the role that health professionals can play in Facebook-based groups to promote access to information and resources or programs. For example, health professionals might be engaged in addressing concerns regarding the potential disclosure of sensitive or dangerous information related to the care of vulnerable children or youth or the caregivers themselves.

Finally, the degree to which Facebook groups can be customized to the specific needs of a target population requires further investigation. This study involved a Facebook group created by a research team in response to an identified gap [32]; further examination of how such groups potentially differ from those created by caregivers or patients themselves is needed. This future work might consider engaging children or youth in a patient-oriented approach to determine how web-based support groups might further address their care needs.

### Conclusions

Patients and caregivers are increasingly engaging in web-based P2P communication to seek and provide support. Investigations into the use of these web-based groups have demonstrated the importance of these communities in meeting the support needs of diverse populations, such as caregivers of children and youth with CCN. P2P support through social media presents a low-cost and accessible avenue for caregivers of children and youth with CCN to obtain needed and timely support. Determining the potential role that health professionals can play in these communities may improve information sharing and the well-being of families of children and youth with CCN.

## Abbreviations

CCN: complex care needs
NB: New Brunswick
P2P: peer-to-peer
PFAC: Patient and Family Advisory Council
